# The small molecule rhodomyrtone suppresses TNF-α and IL-17A-induced keratinocyte inflammatory responses: A potential new therapeutic for psoriasis

**DOI:** 10.1371/journal.pone.0205340

**Published:** 2018-10-15

**Authors:** Julalak Chorachoo, Sylviane Lambert, Teal Furnholm, Liza Roberts, Laura Reingold, Sauvarat Auepemkiate, Supayang P. Voravuthikunchai, Andrew Johnston

**Affiliations:** 1 Department of Dermatology, University of Michigan School of Medicine, Ann Arbor, Michigan United States of America; 2 Department of Microbiology and Excellence Research Laboratory on Natural Products, Faculty of Science and Natural Product Research Center of Excellence, Prince of Songkla University, Hat Yai, Songkhla, Thailand; 3 Department of Pathology, Faculty of Medicine, Prince of Songkla University, Hat Yai, Songkhla, Thailand; NYU Langone Medical Center, UNITED STATES

## Abstract

Psoriasis is a common skin disease pathogenically driven by TNF and IL-17A-induced epidermal hyperproliferation and inflammatory responses. The ongoing need for new therapeutic agents for psoriasis has highlighted medicinal plants as sources of phytochemicals useful for treating psoriatic disease. Rhodomyrtone, a bioactive phytochemical from Rhodomyrtus tomentosa, has well-established anti-proliferative activities. This study assessed the potential of rhodomyrtone for curtailing TNF/IL-17A-driven inflammation. Stimulating human skin organ cultures with TNF+IL-17A to model the skin inflammation in psoriasis, we found that rhodomyrtone significantly decreased inflammatory gene expression and the expression and secretion of inflammatory proteins, assessed by qRT-PCR, immunohistochemistry and ELISA assays respectively. RNA-seq analysis of monolayer primary keratinocytes treated with IL-17A/TNF showed that rhodomyrtone inhibited 724/1587 transcripts >2-fold altered by IL-17A/TNF (p<0.01), a number of which were confirmed at the mRNA and protein level. Suggesting that rhodomyrtone acts by modulating MAP kinase and NF-κB signaling pathways, rhodomyrtone inhibited TNF-induced ERK, JNK, p38, and NF-κBp65 phosphorylation. Finally, assessing the *in vivo* anti-inflammatory potential of rhodomyrtone, we examined its effects on imiquimod-induced skin inflammation in mice, finding rhodomyrtone reversed imiquimod-induced skin hyperplasia and epidermal thickening (*p*< 0.001). Taken together, these results suggest that rhodomyrtone may be useful in preventing or slowing the progression of inflammatory skin disease.

## Introduction

Psoriasis is a common and chronic autoimmune inflammatory skin disorder that has a prevalence of 2–3% in the world’s population [1]. The disease is clinically characterized by red, scaly, and well-demarcated skin lesions that are a consequence of epidermal hyperplasia, altered keratinocyte differentiation, and a dense dermal inflammatory infiltrate. Although the disease pathogenesis is still not fully understood, many lines of evidence suggest that psoriasis epidermal inflammation is mainly driven by activated T cells [2]. Cytokines play a central role in the psoriasis disease process [3], particularly IL-17A and TNF-α, as highlighted by the efficacy of recently-developed biologic drugs targeting these molecules in psoriasis [4, 5]. Despite their efficacy, biologics have a number of significant caveats, including expense, need for injection/infusion, adverse reactions, and loss of efficacy over time [6] thus there is an ongoing need for discovery of new biological targets and novel therapeutics.

Medicinal plants are an important source of biologically active natural products. Downy rose myrtle, *Rhodomyrtus tomentosa*, is an evergreen shrub native to Southeast Asia and been used in traditional medicine to treat a diverse array of diseases [7, 8]. Rhodomyrtone, one of a number of acylphloroglucinol compounds isolated from the leaves of *R*. *tomentosa*, is bioactive and has been demonstrated to possess immuno-modulatory [9] and anti-proliferative properties [10]. We have previously shown that at high concentrations (>2 μg/ml), rhodomyrtone can cause growth arrest and apoptosis of the HaCaT keratinocyte cell line, and inhibit an *in vitro* wound-healing assay. These observations lead us to examine the potential of rhodomyrtone as a therapeutic for treating the skin disease psoriasis, which involves both activation of the immune system and cytokine-driven keratinocyte hyperproliferation [2]. We demonstrate that at low micromolar concentrations (ng/ml range), rhodomyrtone inhibits TNF-α and IL-17A-induced keratinocyte inflammatory responses in both monolayer and organotypic cultures by preventing the activation of key inflammatory signal transduction pathways involving NF-κB and ERK, JNK, and p38 protein kinases following cytokine stimulation. Moreover, we show that topical application of rhodomyrtone can reverse the inflammatory effects of imiquimod on mouse skin, suggesting *in vivo* potential for this compound. Our findings suggest that rhodomyrtone is a candidate for further investigation as a new therapeutic for psoriasis treatment.

## Materials and methods

### Purification of rhodomyrtone

Rhodomyrtone was isolated as described elsewhere [11]. The purity of rhodomyrtone was confirmed by nuclear magnetic resonance and mass spectrometry [12]. A 1 mg/ml stock solution was prepared in DMSO and stored at -20°C until use.

### *Ex-vivo* skin organ cultures

Six-millimeter full-thickness biopsies of normal skin were obtained from healthy volunteers. Informed consent was obtained from all subjects, under protocols approved by the Institutional Review Board of the University of Michigan. This study was conducted in compliance with good clinical practice and according to the Declaration of Helsinki Principles. Biopsies were washed in HBSS (Invitrogen) then bisected twice and incubated in medium M154 supplemented with 1% human keratinocyte growth supplement (HKGS), 1.8 mM CaCl_2_ and penicillin-streptomycin-fungizone (Invitrogen). After rhodomyrtone pretreatment (400 ng/ml) for 6 h, skin biopsies were stimulated with TNF-α (10 ng/ml) and IL-17A (20 ng/ml, both R&D Systems) for 72 h. Skin biopsies were either snap frozen in liquid nitrogen, pulverized, dissolved in complete RLT buffer, and homogenized using glass beads (Biospec Products) for RNA extraction; or fixed overnight in 4% formalin, embedded in paraffin and prepared for immunohistochemistry.

### Immunohistochemistry

Formalin fixed and paraffin embedded skin was sectioned at 5 μm, and then stained using goat anti-human BD-2 (2.5 μg/ml; Peprotech), rat anti-human IL-1F9 (1 μg/ml; Santa Cruz Biotechnology), and mouse anti-human S100A7 (1 μg/ml; R&D Systems). Subsequently, the appropriate species of secondary detection kit was used (Vectorstain ABC; Vector Laboratories) followed by visualization with 3,3’-DAB substrate (BD Pharmingen) and counterstaining with CAT hematoxylin (Biocare Medical). Appropriate isotype control antibodies were used at the same concentrations as the primary detection antibodies and gave no discernable staining.

### ELISAs

CXCL1 ELISA was performed with a DuoSet kit from R&D Systems. Estimation of HBD-2 was carried out as previously described [13].

### Keratinocyte cultures

Primary normal human keratinocyte (NHK) cultures were established from adult human skin as described [14] in serum-free medium M154 (Invitrogen) with 0.1 mM CaCl_2_, penicillin-streptomycin (Invitrogen), 1% HKGS, and incubated at 37°C with 5% CO_2_. NHK were used for experiments in the second or third passage, seeded at 5,000 cells/cm^2^ and maintained to 4-days post confluence. Cultures were then starved of growth factors in unsupplemented medium M154 for 24 h before use. NHK were pretreated with rhodomyrtone (50–600 ng/ml) for 1 h before stimulation with recombinant human 20ng/ml IL-17A and 10 ng/ml TNF-α (both R&D Systems) for 24 h. Cells were harvested for RNA isolation in complete RLT buffer (Qiagen) or protein isolation in complete RIPA buffer. Conditioned media were collected and stored at -20°C and used for estimation of secreted proteins by ELISA.

### RNA-seq

Total RNA was isolated as described above. Ribosomal RNA was removed with a Ribozero kit (Illumina). RNAseq libraries were prepared using a TruSeq Stranded mRNAseq Sample Prep kit (Illumina). The libraries were quantitated by qPCR and sequenced on a HiSeq 4000 sequencer. Unprocessed fastq files were mapped to exons from the human genome (GENCODE release 27), using STAR (v2.5.2a) with default settings and a maximum of 1 hit per read. Read counts for reads with a minimum length of 50 were obtained using the STAR—quantMode GeneCounts setting. A read count matrix was generated using an in-house R script, and voom-limma.R package was used for read normalization and gene expression analysis [15]. The t-tests relative to a threshold (TREAT) method [16] with 2-fold expression change threshold was applied to the limma empirical Bayes model to generate the FDR controlled differentially expressed gene list. Gene otology (GO) analysis was performed with ClueGO version 2.5.0 plugin for CytoScape 3.6.0. Gene Set Enrichment Analysis was performed using an in-house R script as described previously by our group [17].

### RNA isolation and quantitative real-time RT-PCR

RNA was extracted using RNeasy plus mini kit (Qiagen) and 1 μg total RNA reverse transcribed (Taqman Reverse Transcription kit; Applied Biosystems). Each reaction was performed with cDNA reverse transcribed from 10 ng RNA. qRT-PCR was performed using TaqMan Gene Expression Master Mix with TaqMan primers and fluorescent probes from Applied Biosystems for *CXCL1* (Hs00605382_gl), *DEFB4B* (Hs00175474_ml), *IL36G* (Hs00219742_ml), *IL36RN* (Hs00202179_ml), *LCN2* (Hs00194353_ml), *MMP13* (Hs00233992_ml), *PI3* (Hs00160066_ml), *S100A7* (Hs00161488_ml), *S100A8* (Hs00374264_gl), and *S100A9* (Hs00610058_ml) on an Applied Biosystems Fast PCR machine. Target gene levels were normalized to the expression of the housekeeping gene *RPLP0* (Hs99999902_ml).

### Western blotting

After rhodomyrtone pretreatment (600 ng/ml, 24 h), NHK were stimulated with TNF-α (10 ng/ml) for 0, 10, 20, 30, and 60 min. Cells were lysed in 200 μl Laemmli buffer, and lysates boiled at 95°C for 5 min. Proteins were separated on 4–20% SDS gels and transferred to Immobilon-P membranes, followed by blocking for 1 hour then incubation with primary antibodies (dilution 1:1,000; Cell Signaling) overnight at 4°C. Membranes were washed with TBS +0.1% Tween and incubated with secondary antibodies (dilution 1:2,500; Cell Signaling) for an hour at room temperature. Membranes were scanned using Image Studio Lite v3.1.Ink (LI-COR). For control of equal protein loading, the membrane was normalized with antibody against β-actin (dilution 1:2,500; Sigma).

### Animal experiments

Male ICR mice (6–8 weeks, 25-35g) were acclimated to standard housing and feeding. All methods were performed in accordance with the guidelines and regulations approved by the animal ethics committee, Prince of Songkla University, Hat Yai, Thailand. Animals were housed in individual cages under specific pathogen-free conditions with 12 h dark/light cycle and provided with food and water. Skin inflammation in mice was induced by daily topical application of a dose of 62.5 mg of 5% imiquimod (IMQ) cream (Aldara) on the shaved back skin for 6 consecutive days. After exposure to IMQ, fifty-four mice were divided into six groups: untreated (negative control), base formulation without rhodomyrtone (vehicle control), IMQ alone (positive control), 0.181 mg/cm^2^ rhodomyrtone, 0.364 mg/cm^2^ rhodomyrtone, and 0.0155 mg/cm^2^ betamethasone treated. All animals were housed for a total period of 15 days, and skin biopsies taken every 3 days. At the end of the experiments, animals were anaesthetized deeply with thiopental sodium and sacrificed by cervical dislocation. The experimental design for the induction of IMQ-induced skin inflammation and subsequent treatment studies in mice is summarized in S4 Fig.

### Histopathological analysis

Mice were anesthetized with thiopental sodium (90 mg/kg) by intra-peritoneal injection. The dorsal area of each mouse was completely shaved and sterilized with 70% ethanol. Lidocaine (2%) was applied as a topical anesthetic and skin pieces were excised using a 4 mm biopsy punch. Skin sections were taken and immediately fixed with 10% formalin solution, dehydrated with a graded ethanol series, and embedded in paraffin. Skin tissue samples (n = 9/group) after 6, 9, 12, and 15 days of treatment were subjected to histological analysis stained with haematoxylin-eosin. The thickness of epidermis (from the basal layers to stratum corneum) was determined by measuring thickness in more than 10 fields, at intervals of 4 mm in each section using computing software (ImageJ1.42q/Java1.6.0 10).

### Statistical analysis

All tested groups were compared with the control groups or TNF-α treatment alone and the results were analyzed statistically using one-way ANOVA and Dunn’s multiple comparison test. Continuous variables were described in terms of mean and standard deviation. All calculations were performed with GraphPad Prism v7.00. The values of *p*<0.05 were considered to be statistically significant.

## Results

### Development and validation of *ex vivo* skin organ cultures

**Fig 1:**
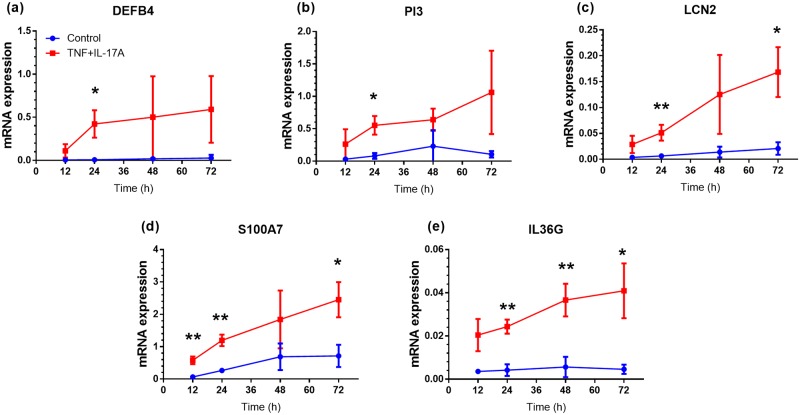
TNF-α and IL-17A drive psoriasis transcript expression in *ex vivo* skin organ cultures. To validate the use of cytokine-stimulated normal human skin biopsies as a model of skin inflammation, we treated biopsies with a combination of 10 ng/ml TNF-α and 20 ng/ml IL-17A for 12, 24, 48, and 72 h and monitored expression of *DEFB4*, *PI3*, *LCN2*, *S100A7*, and *IL36G* transcripts by qRT-PCR. Values are mean ± SD of results in triplicate from 3 donors. Statistical significance indicated by *p<0.05, **p<0.01, as compared with untreated cultures at each time point.

We developed an *ex vivo* organotypic skin culture model using full-thickness normal human skin stimulated with IL-17A and TNF-α to mimic disease-related molecular changes in psoriasis skin. From preliminary experiments we determined that medium M154 supplemented with 1% HKGS and 1.8 mM calcium chloride was optimal for short-term (up to 72 h) culture of normal human skin biopsies. These cultures were treated with a combination of 20 ng/ml IL-17A and 10 ng/ml TNF-α to model some of the inflammatory processes seen in psoriasis skin[18]. Recapitulating psoriasis skin, IL-17A+TNF-α treatment lead to increased expression of *DEFB4*, *PI3*, *LCN2*, *S100A7*, and *IL36G* mRNA over a 72 h time course (Fig 1) without marked deterioration of tissue structure (S1 Fig).

### Rhodomyrtone inhibits cytokine-induced inflammatory responses in *ex-vivo* skin organ cultures

**Fig 2:**
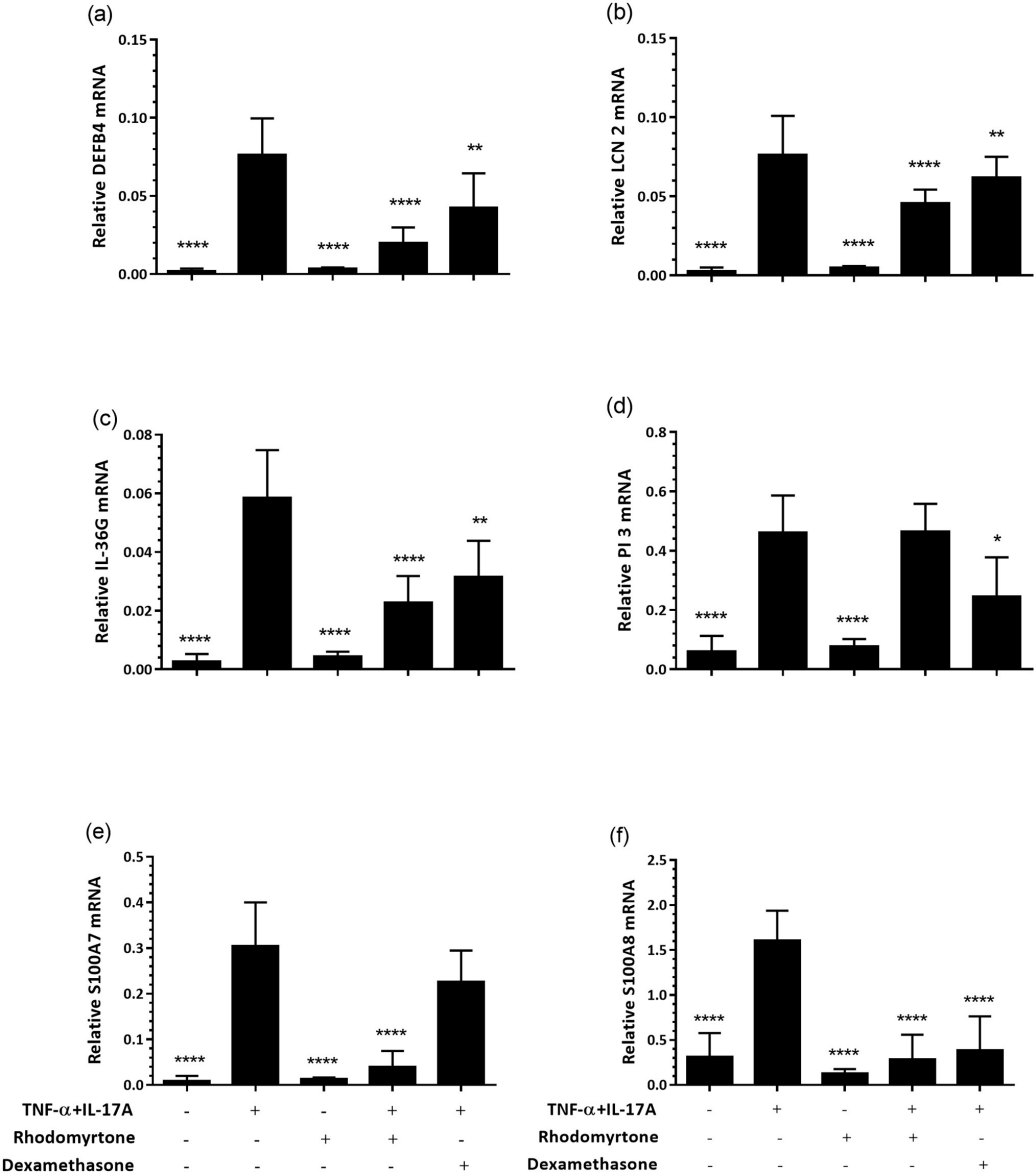
Rhodomyrtone decreases expression of inflammatory transcripts by TNF-α+IL-17A stimulated in skin organ cultures. Skin biopsies were pre-treated with 400 ng/ml rhodomyrtone 6 h before stimulation with 10 ng/ml TNF-α and 20 ng/ml IL-17A. Rhodomyrtone treatment decreases production of *DEFB4*, *IL1B*, *IL17C*, *IL36G*, *LCN2*, *PI3*, *S100A7*, and *S100A8* transcripts as measured by qRT-PCR at 72 h. Statistical significance determined by one-way ANOVA and Dunn’s multiple comparison test, indicated as *p<0.05, **p<0.01, ****p<0.0001 versus TNF-α+IL-17A treatment.

To determine whether rhodomyrtone has potential for countering psoriatic skin inflammation we assessed the effects of rhodomyrtone on our *ex vivo* skin inflammation model. We pre-treated skin tissues with 400 ng/ml rhodomyrtone, the highest dose shown to have no toxicity (S2 Fig), for 6 h before stimulation with TNF-α and IL-17A. After 72 h, tissues were harvested and prepared for qRT-PCR, immunohistochemistry and conditioned media collected. Results from qRT-PCR measurements of psoriasis-relevant transcripts showed that levels of *DEFB4B*, *IL36G*, *LCN2*, *S100A7*, *S100A8* mRNA were significantly decreased in rhodomyrtone-treated cultures when compared with cytokine-treated cultures (Fig 2, *p*<0.0001, all). Similar results were observed with anti-inflammatory steroid (dexamethasone) treatment.

**Fig 3:**
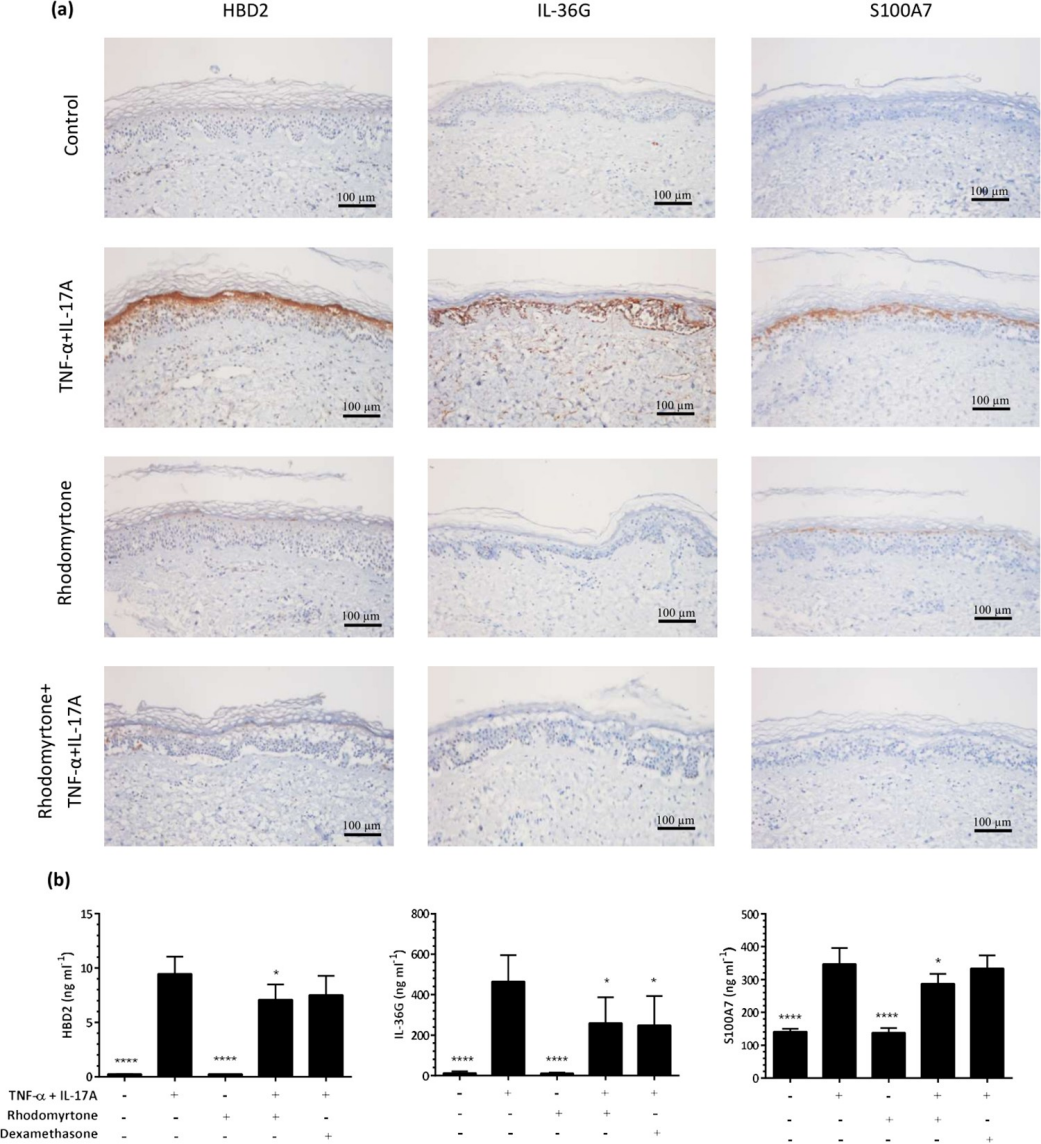
Rhodomyrtone inhibits TNF-α+IL-17A-induced inflammatory responses in skin organ cultures. Cultures were pre-treated with 400 ng/ml rhodomyrtone for 6 h before stimulation with 10 ng/ml TNF-α and 20 ng/ml IL-17A for 72 h. Immunohistochemical staining for HBD-2, IL-36γ, and S100A7 revealed prominent induction of all three proteins by TNF+IL17A treatment (a). ELISA of conditioned media at 72 h confirmed the induction of HBD-2, IL-36γ, and S100A7 by IL17A+TNF (b). Staining from 1 representative donor of 5 shown, staining from 4 others shown in S2 Fig. Bars indicate mean ± SD, *n* = 5. Statistical significance determined by one-way ANOVA and Dunn’s multiple comparison test, indicated as*p<0.05 versus TNF-α and IL-17A treatment alone. Scale bar, 100 μm.

The effects of rhodomyrtone on skin inflammatory responses were also assessed in terms of human β-defensin (HBD)-2, IL-36γ, and S100A7 protein expression in skin sections using immuno-histochemistry. HBD-2, IL-36γ, and S100A7 protein expression was strongly upregulated following treatment with TNF-α+IL-17A (Fig 3a and S3 Fig). Although rhodomyrtone treatment alone had little to no effect, rhodomyrtone curtailed the cytokine-induced increase in the expression of all three of these proteins to the extent that the expression of HBD-2, IL-36γ, and S100A7 was at a level observed in untreated skin specimens (Fig 3 and S3 Fig). Corroborating our immunohistochemical findings, measurement of secreted HBD-2, IL-36γ, and S100A7 proteins using ELISA revealed TNF-α+IL-17A-treated skin cultures elaborated significantly increased amounts of the HBD-2, IL-36γ, and S100A7 into the media (Fig 3b, *p*<0.0001, all) which rhodomyrtone significantly inhibited by 25%, 45%, and 17% respectively (*p*<0.05, all). Interestingly, dexamethasone reduced cytokine-induced IL-36γ production but had no significant inhibitory effects on HBD-2 and S100A7 secretion.

### Rhodomyrtone inhibits IL-17A/TNF-α-induced keratinocyte inflammatory responses

**Fig 4:**
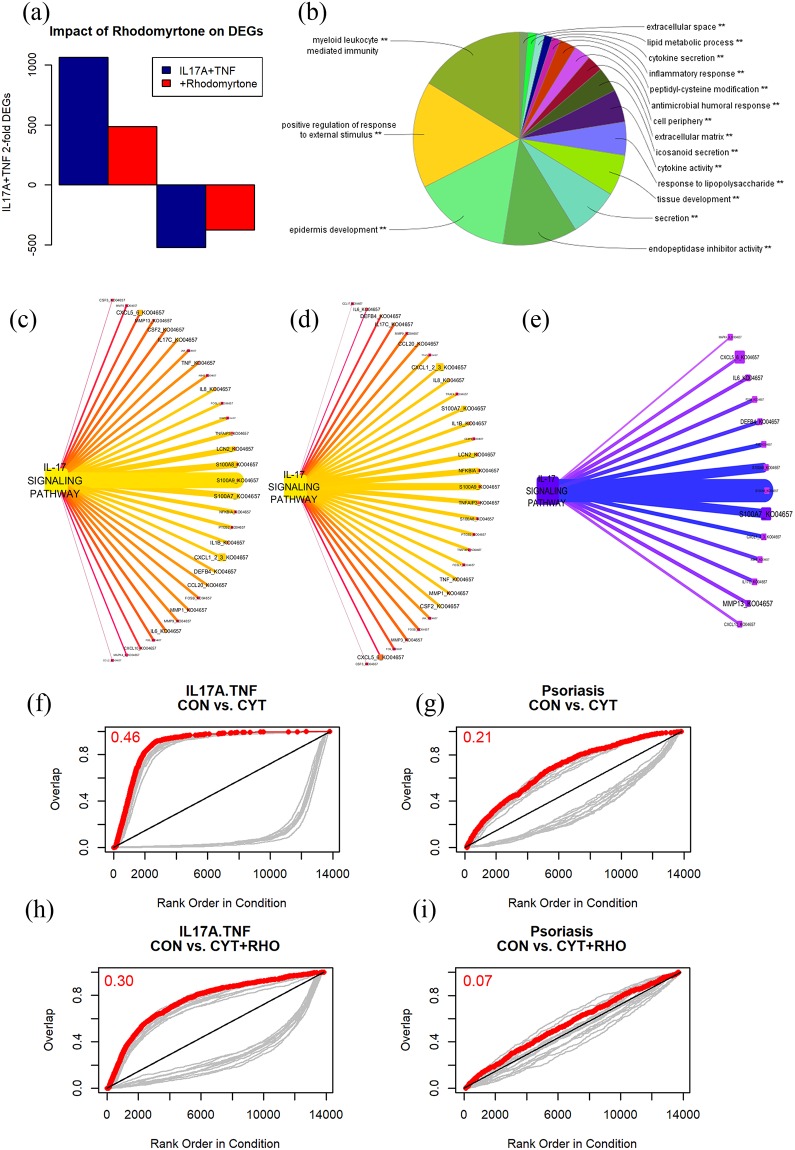
Rhodomyrtone preferentially down-regulates transcripts involved in epidermal responses, myeloid leukocyte chemotaxis and inflammatory responses. Global transcriptome sequencing (RNA-seq) was performed on NHK treated with IL-17A+TNF for 24 h with or without 1 h pre-treatment with 400 ng/ml rhodomyrtone. Rhodomyrtone inhibited the expression of 579 of 1066 IL-17A/TNF-induced genes by NHK (2-fold change, TREAT *p*<0.01 [16]) and increased the expression of 145/521 IL-17A/TNF-suppressed genes (a). Gene ontology (GO) [19] analysis of these DEGs revealed suppression of epidermal responses, myeloid leukocyte chemotaxis and inflammatory response pathways (e). KEGG pathway analysis of the DEGs revealed that while the cytokine vs control (c) and cytokine+rhodomyrone vs control (d) comparisons shared many of the same up-regulated gene functions, there was an overall reduction in DEG expression levels and/or number of DEGs per KEGG function (e.g. CXCL1) in the IL-17 pathway when cytokine exposed cells were pretreated with rhodomyrtone. These shifts in gene expression were highlighted when cytokine+rhodomyrtone transcripts were compared against cytokine-only transcripts (e). Node color and font size is the sum of the log2 fold-change of either the up-regulated or down-regulated DEGs. The line color and thickness is the sum of averaged DEG read counts across the 3 samples of either the numerator condition (up-regulated) or denominator condition (down-regulated). Gene set enrichment analysis (GSEA) [17] showed a decrease in the expression of IL-17/TNF induced transcripts area under curve = 0.46 (f) vs. 0.30 (h) and a decrease in genes overlapping with a plaque psoriasis gene signature, area under curve = 0.21 (g) vs. 0.07 (i).

To better understand the inhibitory effects of rhodomyrtone on cytokine stimulated KC, we treated NHK with IL-17A (20 ng/ml) + TNF-α (10 ng/ml) for 24h and monitored gene expression by global transcriptome sequencing (RNA-seq). RNA-seq produced an average of 30 million 100 nt reads/sample (GEO accession #TBA), which aligned to 26,289 human genes. Compared with untreated control cells, 1588 differentially expressed genes (DEGs) were detected after IL-17A/TNF treatment (fold change ≥±2, TREAT *p*<0.01 [16]) with 1066 up- and 521 down-regulated). Rhodomyrtone significantly suppressed the expression of 579/1066 IL-17A/TNF-induced DEGs and increased the expression of 145/521 IL-17A/TNF-suppressed DEGs (Fig 4a and S1 Table). Gene ontology (GO) analysis [19] revealed these DEGs were enriched for genes involved in leukocyte/granulocyte chemotaxis, epidermal development and inflammatory responses (Fig 4b). In addition, KEGG [20] pathway analysis revealed decreased expression of IL-17 pathway-associated genes including S100A7, DEFB4, S100A8, S100A12, MMP13, IL17C, CXCL1, IL6 (Fig 4c–4e). This down-shift of IL-17A/TNF induced genes was also highlighted using gene set enrichment analysis (GSEA) [17] that showed a weaker IL-17A/TNF gene signature when cytokine-treated cells (Fig 4f) were treated with rhodomyrtone (Fig 4h). GSEA also highlighted the presence of a psoriasis gene signature in the IL-17A/TNF-treated cells (Fig 4g) which was suppressed by treatment with rhodomyrtone (Fig 4i).

**Fig 5:**
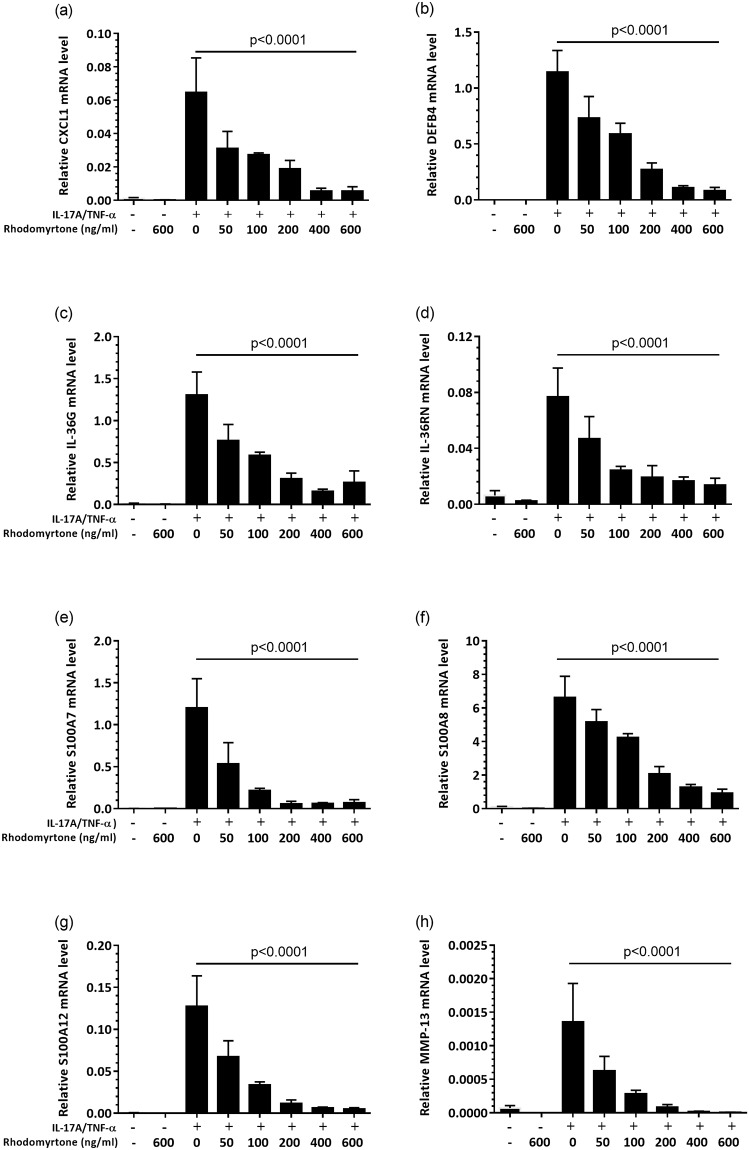
Rhodomyrtone dose dependently decreases production of *CXCL1*, *IL-36G*, *IL-36RN*, *MMP-13*, *S100A7*, *S100A8*, and *S100A12* transcripts by IL-17A/TNF-α-stimulated primary human keratinocytes. Keratinocytes were pretreated with rhodomyrtone (50, 100, 200, 400, and 600 ng/ml) before stimulation with 20ng/ml IL-17A+10ng/ml TNF-α. The mRNA expression levels were measured at 24 h by qRT-PCR. Values are mean±SD of results from three independent experiments in triplicate. Statistical significance determined using one-way ANOVA and Dunnett’s multiple comparison test and noted ****p<0.0001, as compared with IL-17A/TNF-α treatment.

**Fig 6:**
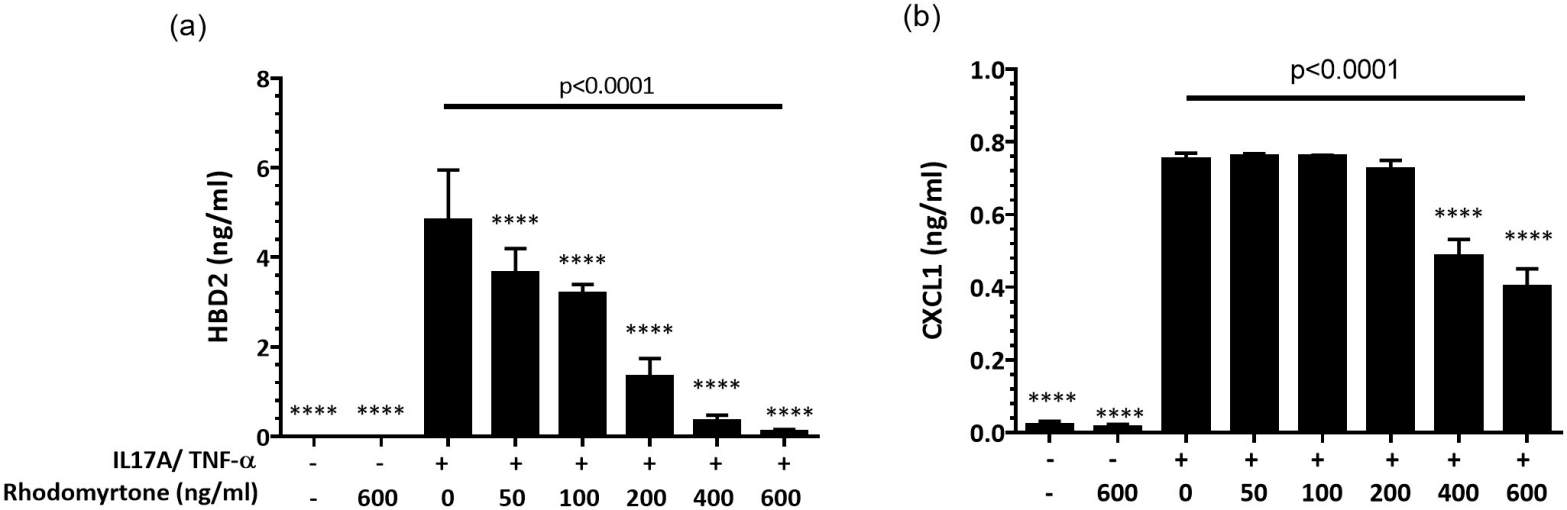
Rhodomyrtone inhibits IL-17A/TNF-α-induced inflammatory responses in keratinocytes. Primary human keratinocytes were pretreated with rhodomyrtone (50, 100, 200, 400, and 600 ng/ml) for 1 h before stimulation with 20 ng/ml IL-17A+10 ng/ml TNF-α. HBD-2 (a), and CXCL-1 (b), were assayed in conditioned media at 24 h by ELISA and found to be reduced in a dose-dependent manner in the presence of rhodomyrtone. Values are mean ± SD of results from three independent experiments in triplicate. Statistical significance determined using one-way ANOVA and Dunnett’s multiple comparison test and noted ****p<0.0001, as compared with TNF-α treatment alone.

These changes in gene expression were confirmed with qRT-PCR, where pretreatment with rhodomyrtone (0.125–1.5 μM, 50–600 ng/ml) 1 h before stimulation prevented IL17A+TNF-induced expression of *CXCL1*, *DEFB4*, *IL36G*, *IL36RN*, *S100A7*, *S100A8*, *S100A12*, and *MMP13* by keratinocytes (Fig 5). The inhibitory effects of rhodomyrtone on IL-17A+TNF-α-induced inflammatory response were also evident at the protein level. NHK pretreated with rhodomyrtone (50–600 ng/ml) secreted significantly less CXCL1, and HBD-2 (all, *p*<0.0001, ANOVA) into the conditioned media at 24 h in a dose-dependent manner as measured by ELISA (Fig 6).

### Rhodomyrtone regulates the TNF-α-induced activation of the MAPK and NF-κB signaling pathways

**Fig 7:**
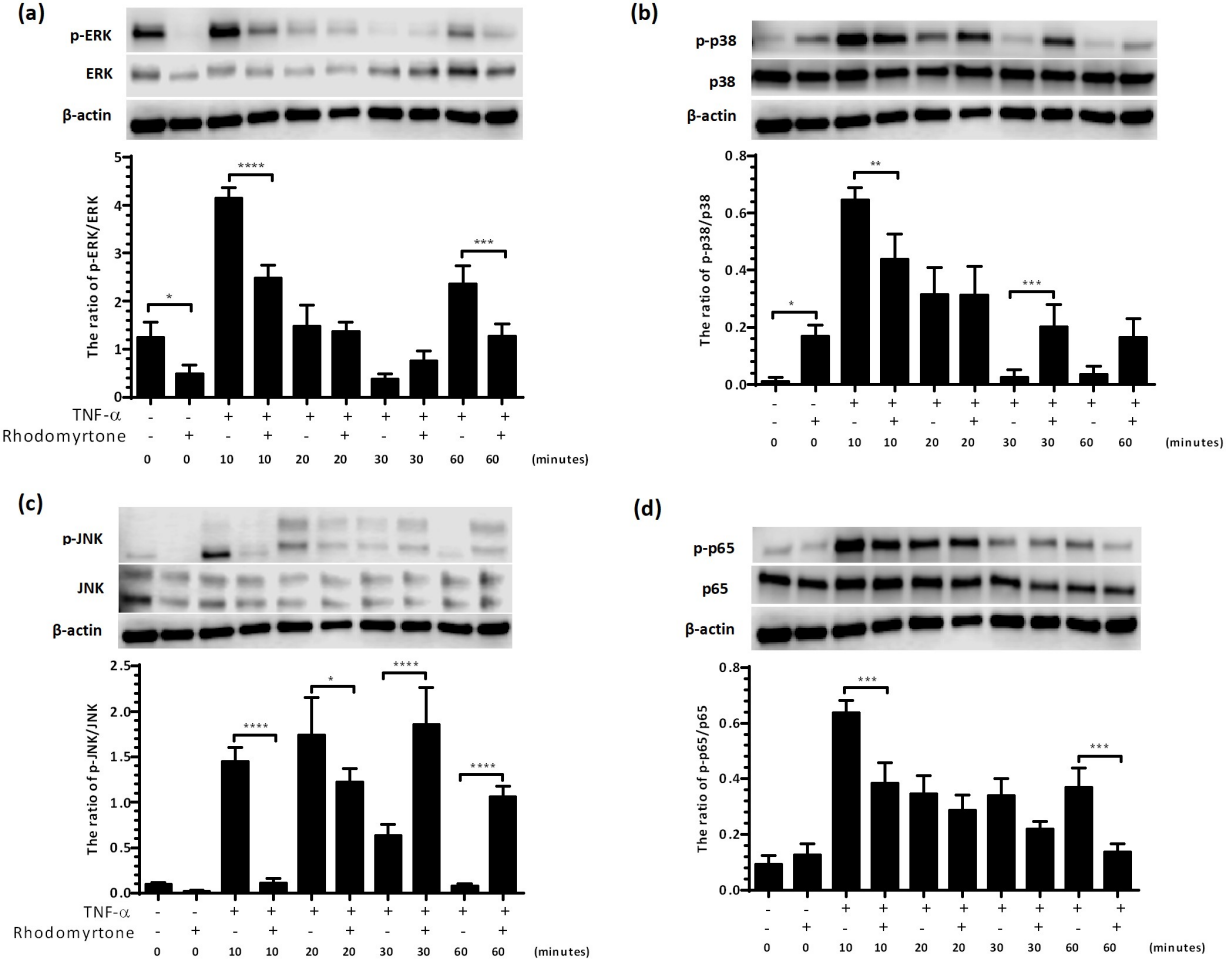
Rhodomyrtone suppressed the TNF-α induced phosphorylation of MAPKs and NF-κBp65 in primary human keratinocytes. Keratinocytes were pre-incubated with 600 ng/ ml rhodomyrtone for 24 h and stimulated with TNF-α for 0, 10, 20, 30, and 60 min. The ratio of p-ERK/ERK (a), p-p38/p38 (b), p-JNK/JNK (c), and p-p65/p65 (d) was analyzed by western blot analysis, and protein expression was quantitated using Image Studio Lite v5.2.Ink software and normalized to β-actin. Values are mean ± SD of results from three independent experiments in triplicate. (*p<0.05, **p<0.01, ***p<0.001, ****p<0.0001, as compared with TNF-α at each time point).

Given that rhodomyrtone inhibited TNF and IL-17A-induced inflammatory responses, and these responses are dependent on MAPK and NF-κB signaling, we next examined these pathways to elucidate how rhodomyrtone inhibits inflammatory responses. We treated NHK cultures with 600 ng/ml rhodomyrtone for 24 h before stimulating with 10 ng/ml TNF-α and examined the effects of rhodomyrtone on TNF-α-induced phosphorylation of MAPKs and NF-κBp65 at 10, 20, 30, and 60 min by western blotting (Fig 7). At 10 min, TNF-α clearly increased the levels of ERK, JNK, and p38 phosphorylation (normalized to total JNK, p38, and ERK, respectively) compared with untreated control cells, and this TNF-α-induced ERK (Fig7a), p38 (Fig 7b), and JNK (Fig 7c) phosphorylation was significantly inhibited by pretreatment with rhodomyrtone. In contrast, at 30 and 60 min of TNF stimulation, pretreatment with rhodomyrtone significantly increased TNF-α-induced phosphorylation of JNK, p38 but not ERK. In addition to its effects on TNF-induced MAPK activation, rhodomyrtone suppressed NF-κBp65 phosphorylation which was significantly suppressed by treatment with 600 ng/ml rhodomyrtone at 10 and 60 min (normalized to total NF-κBp65, Fig 7d) with no discernable effect on NF-κBp65 activation without TNF-α-stimulation. These results suggest that rhodomyrtone exhibits anti-inflammatory effects by inhibiting the production of pro-inflammatory mediators via inhibition of the MAPK and NF-κB signaling pathways.

### Effects of rhodomyrtone imiquimod-induced psoriasiform skin inflammation in mice

**Fig 8:**
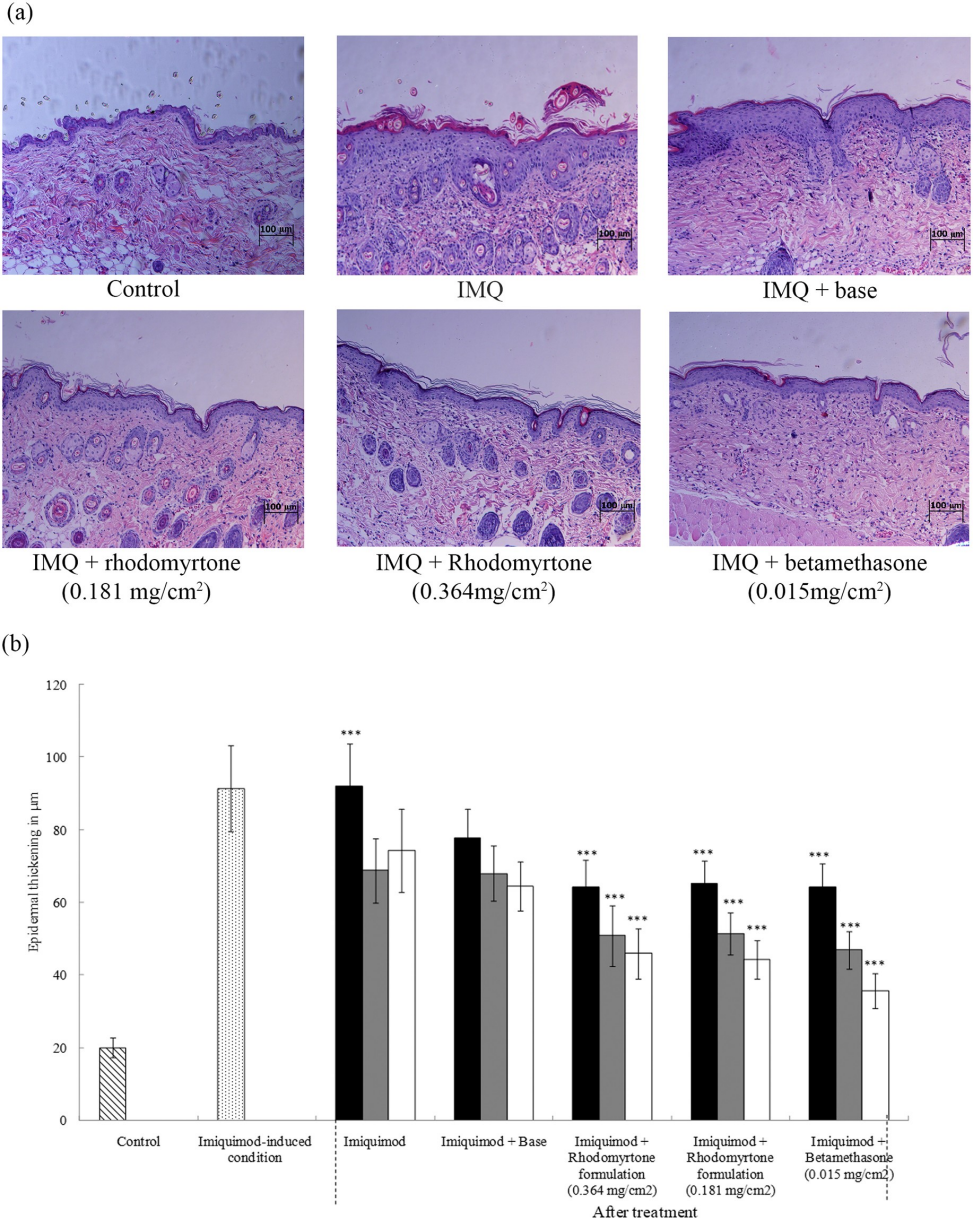
Rhodomyrtone attenuates imiquimod (IMQ)-induced skin inflammation in mice. Mice were treated for 6 consecutive days with IMQ on the shaved back. Betamethasone (0.0155 mg/cm^2^), rhodomyrtone (0.181 mg/cm^2^), rhodomyrtone (0.364 mg/cm^2^), IMQ, and vehicle were topically administered for 15 days. A skin lesion biopsy was removed and fixed in 10% (v/v) paraformaldehyde for 3 (■), 6 (■), and 9 (□) days. Representative H&E-stained sections of skin after 15 days of treatment (a) and epidermal thickness of the back skin was measured in 10 subsequent areas in μm and the mean was calculated (b). n = 9 each group, ***p<0.001, as a significant compared with vehicle-only.

To begin to assess the in vivo anti-inflammatory potential of rhodomyrtone, we used the imiquimod (IMQ)-induced skin inflammation mouse model [21]. Daily topical application of 62.5 mg IMQ per mouse for 6 days resulted in the development of psoriasiform inflammation on the dorsal portion of mice with the development of erythema, scales, and skin thickening after three days of exposure to IMQ (Fig 8a and S5 Fig). In contrast, mice topically treated with rhodomyrtone formulations remained smooth with no overt signs of inflammation (Fig 8a and S5 Fig), compared with base formulation which appeared to have a partial effect on reducing psoriasis-like inflammation. The efficacy of the rhodomyrtone formulation was comparable to the clinically-used topical steroid, betamethasone (Fig 8). Histological analysis of H&E-stained IMQ-treated back skin confirmed the increase in epidermal thickness, which appeared to be due to the basal keratinocyte hyperplasia, epidermal acanthosis, and perivascular infiltration of inflammatory cells in the upper dermis similar to that seen in psoriasis skin lesions. Rhodomyrtone formulations reduced the IMQ-induced skin inflammation as evidenced by a decrease in epidermal thickening (Fig 8a and 8b). By day 15, the basket-weave pattern of the cornified layer and the compact orthokeratosis of normal skin were observed in both rhodomyrtone formulations tested as well as the betamethasone control. No abnormal phenotype was observed in the control groups. The observed maximum increase in back skin thickness in IMQ-treated mice was 91.39 ± 11.84μm on day 6 (Fig 8b) and it was at this point that we added rhodomyrtone (0.181 and 0.364 mg/cm^2^) or betamethasone (0.0155 mg/cm^2^) to the treatment regimen. Application of rhodomyrtone at day 6 of IMQ-treatment was effective in significantly reducing epidermal thickness when measured on days +3, +6 and +9 at both concentrations used (0.181 and 0.364 mg/cm^2^ rhodomyrtone) and this was as effective as betamethasone treatment at all time points tested (Fig 8b). Thus, topical rhodomyrtone could reverse IMQ-induced skin inflammatory responses in this *in vivo* model.

## Discussion

Psoriasis lesions contain elevated levels of TNF-α [22] and IL-17A, cytokines recognized as important mediators of cutaneous inflammation [18, 23, 24]. Biologics targeting TNF-α [4, 5] and IL-17A [25, 26] have been developed and found to be very effective for managing psoriasis. Despite their efficacy, these biologics have a number of drawbacks and side effects. Because of their high molecular weight, biologics have to be administered by injection or infusion and are expensive to produce. Biologics can lose efficacy over time and may trigger a number of side-effects including speckled hyperpigmentation [27], increased risk of infection [6, 28], as well as the development of secondary autoimmune diseases including cutaneous vasculitis, hematological diseases, and other autoimmune diseases [29, 30]. Therefore, there remains a need for the development of effective and inexpensive oral or topical psoriasis treatments.

Recently, there has been a resurgence of interest in the potential of phytochemicals as sources of small molecules for treating inflammatory diseases. Traditionally-used plants are a source of a varied array of bioactive small molecules which may yield promising lead compounds for psoriasis treatment. Recent examples that have been investigated and shown anti-inflammatory effects include resveratrol [31], a stilbenoid isolated from grapes, nuts, and berries, and paeoniflorin [32], amonoterpene glycoside from *Paeonia lactiflora*. Rhodomyrtone is a bioactive acylphloroglucinol from *Rhodomyrtus tometosa* which has previously been shown to have immuno-modulatory [9] and anti-proliferative effects [10].

To model the cytokine-induced keratinocyte responses seen in psoriasis, we treated human skin organ cultures with TNF-α and IL-17A. Rhodomyrtone drove significant reductions in *DEFB4*, *IL36G*, *LCN2*, *S100A7*, and *S100A8* mRNA expression, and HBD-2, IL-36γ and S100A7 protein expression both in tissues and secreted into culture media by TNF-α+IL-17A-stimulated skin organ cultures (Figs 2 and 3). Although studying keratinocyte and explant cultures cannot fully reflect all of the processes ongoing in psoriatic lesions, such cultures are very useful for modelling and testing specific processes and pathways shown to be present in psoriatic disease, and as such these data demonstrate that rhodomyrtone can suppress keratinocyte inflammatory responses to IL-17A and TNF-α, potentially a useful clinical approach for the treatment of inflammation and psoriasis.

RNA-seq data from NHK treated with TNF and rhodomyrtone confirmed the inhibitory effect of rhodomyrtone, particularly on the IL-17A/TNF induction of DEFB4, IL36G, and CXCL1, all known to be highly expressed in psoriasis lesions and investigated as potential biomarkers [33, 34] (Fig 4 and S1 Table). Confirming our RNA-seq data, we found rhodomytone dose dependently suppressed IL-17A/TNF-α-stimulated expression of a range of inflammatory mediators including the cytokines IL-36γ and IL-36Ra; chemokine CXCL1; matrix metalloproteinase MMP-13 as well as S100A7, S100A8, and S100A12 expression (Fig 5). Corroborating this, we also observed significant decreases in the secretion of CXCL1, HBD-2 proteins (Fig 6).

Intracellular signals for many of the cytokines elevated in psoriasis lesions, including TNF and IL-17A, are transduced though the MAP kinase and NF-κB pathways [3]. Activation of these pathways has been associated with a range of diseases [35] and as such, activated NF-κBp65 has been detected abundantly in lesional psoriasis skin while being absent from normal epidermis [36]. These signal transduction pathways regulate cellular proliferation, differentiation, apoptosis, and inflammation, and as such have been assessed as major therapeutic targets in inflammatory and autoimmune diseases [37–40]. We explored which of these pathways was targeted by rhodomyrtone treatment and found that rhodomyrtone suppressed TNF-α-induced activation of NF-κB as well as ERK, JNK, and p38 MAPK signaling in NHK, suggesting that inhibition of all three major MAPK subfamilies and NF-κB may be involved in the anti-inflammatory effects of rhodomyrtone. Given that the expression of many inflammatory cytokines is downstream of NF-κB and MAPK activation, and that much cross-talk exists between these pathways, such inhibition may give rise to broad anti-inflammatory actions.

We previously showed that at doses at or above 2 μg/ml (2.26 μM) rhodomyrtone caused growth arrest and apoptosis of the HaCaT keratinocyte cell line, and inhibited cell migration [10]. Likewise here we show that at concentrations above 1μg/ml rhodomyrtone induced NHK apoptosis (S2 Fig), and examination of JNK and p38 MAPK phosphorylation at later time-points (Fig 7) suggests that at higher doses, rhodomyrtone induces JNK and p38 phosphorylation potentially driving a JNK-caspase 9 apoptotic program (S6 Fig) consistent with earlier reports [41]. At lower concentrations, however, rhodomyrtone has no discernable effect on cell viability (S2 Fig) whilst inhibiting TNF/IL-17A-induced signaling (Figs 2–6).

Although not a perfect model for human psoriasis, IMQ-induced mouse skin inflammation shares activation of the IL-23/IL-17A axis [42] dependency upon IL-22 [43]and IL-36 [44] to develop lesions as well as an inflammatory leukocytic infiltrate [45]. Our data demonstrated that rhodomyrtone formulations reduced skin inflammation in IMQ-induced animals.

Our findings demonstrate that rhodomyrtone is capable of inhibiting the transcription and expression of a number of inflammatory mediators and antimicrobial peptides induced by TNF-α and/or IL-17A, via suppression of NF-κB, ERK, JNK, and p38 signaling pathways. Additionally, our *in vivo* data support the efficacy of topical rhodomyrtone for treating psoriasis through the inhibition of keratinocyte hyperproliferation. Thus, taken together, our data suggest that rhodomyrtone has potential for further development a sa therapeutic agent against psoriasis and possibly other inflammatory cytokine-mediated diseases.

## Supporting information

S1 Fig Histology of normal human skin organ cultures. Unstimulated skin organ cultures (a), 10ng/ml TNF + 20 ng/ml IL-17A treated skin organ cultures (b). 12–72 hour time course, 3 healthy donors, Scale bar, 100 μm.
(PDF)

S2 Fig Rhodomyrtone toxicity is limited to high doses. Cultures of primary normal human keratinocytes (NHK) were treated with the indicated concentrations of rhodomyrtone or 5μM erlotinib for 24 hours and the level of cell death assessed using 4',6‐diamidino‐2‐phenylindole (DAPI) to identify the sub‐G1 cell cycle population (a and b) and annexin V‐FITC and propidium iodide (PI) staining to identify apoptotic and necrotic cells (c and d) using flow cytometry. Treatment with the EGFR inhibitor erlotinib, a positive control for keratinocyte apoptosis, increased the proportion of keratinocytes in the sub‐G1 (9.8%) as did treatment with 800 and 1,000 ng ml‐1 of rhodomyrtone (27.86% and 37.26%, respectively compared with 2.43% in control cultures); however, at concentrations of 600 ng/ml and below, rhodomyrtone did not exhibit toxicity (a and b). This result was corroborated using annexin V and PI staining which showed that below 600 ng/ml, rhodomyrtone did not induce keratinocyte apoptosis, however, cell death was evident at higher concentrations (c and d). Bar graphs, mean±SD of results from three independent experiments in triplicate. Statistical significance determined using one‐way ANOVA and Dunn’s multiple comparison test and denoted ** p<0.01, *** p<0.001, ****, p<0.0001.
(PDF)

S3 Fig Rhodomyrtone inhibits TNF-α+IL-17A-induced inflammatory responses in skin organ cultures. Cultures were pre-treated with 400ng ml-1 rhodomyrtone for 6 hours before stimulation with 10ng/ml TNF-α and 20ng/ml IL-17A for 72 hours. Immuno-histochemical staining for HBD-2, IL-36γ, and S100A7 revealed prominent induction of HBD-2 (a), IL-36G (b) and S100A7 (c) by TNF+IL17A treatment. Staining from 4 donors of 5 shown. Scale bar, 100 μm.
(PDF)

S4 Fig Experimental procedure of IMQ‐induced skin‐inflammation in mice. Mice received a daily topical dose of 62.5 mg IMQ cream (5% on the shaved dorsal skin for 15 days. Six days after first sensitization, IMQ‐induced mice were treated with rhodomyrtone formulations (0.181 and 0.364 mg/cm), or vehicle group, or betamethasone (0.015 mg/cm) twice a day for 9 consecutive days. At 24 hours after the final administration, mice were sacrificed and samples were taken.
(PDF)

S5 Fig Rhodomyrtone attenuates imiquimod (IMQ)‐induced skin inflammation in mice. The control mice showed no changes in skin lesions (a). The mice received a daily topical dose of 62.5 mg of 5% IMQ cream demonstrate gross skin folds scaling, thickness, and erythema (b). Some decrease in scaling, thickness, and erythema were noticed in mice skin after treatment with base formulation (C). Rhodomyrtone formulation with 0.181 mg/cm (d), 0.364 mg/cm (e), and betamethasone cream with 0.015 mg/cm (f) demonstrated almost complete clearance of scaling, thickness, and erythema on the skin.
(PDF)

S6 Fig At high concentrations, rhodomyrtone triggers activation of keratinocyte apoptosis. HaCaT keratinocytes were treated with 2μg ml-1 rhodomyrtone for 0, 12, 24, 48, and 72 hours then total protein prepared. The effects of rhodomyrtone on expression levels of apoptosis-associated proteins was assesses by western blot analyses using antibodies against Bcl-2, Bid, caspases-7, -8, and -9, and cleaved caspases-7, -8, and -9 (A). Bar graphs represent the ratio of each protein normalized to β-actin expression (B). Values shown are mean ± SD of results from three independent experiments in triplicate. (*p<0.05, **p<0.01, ****p<0.0001, as compared with control).
(PDF)

S1 Table The effects of rhodomyrtone on IL-17A/TNF-mediated differential gene expression as assessed by global transcriptome sequencing (RNA-seq). (XLSX)

S1 Method Apoptosis assay and cell-cycle analysis. (PDF)
