# Supplementary material for: The small molecule rhodomyrtone suppresses TNF-α and IL-17A-induced keratinocyte inflammatory responses: A potential new therapeutic for psoriasis
Source: PLoS One. 2018 Oct 15;13(10):e0205340. doi: 10.1371/journal.pone.0205340 (PMC6188632; doi:10.1371/journal.pone.0205340)
Supplement: S1 Fig — Unstimulated skin organ cultures (a), 10ng/ml TNF + 20 ng/ml IL-17A treated skin organ cultures (b). 12–72 hour time course, 3 healthy donors, Scale bar, 100 μm. (PDF) [file pone.0205340.s001.pdf]

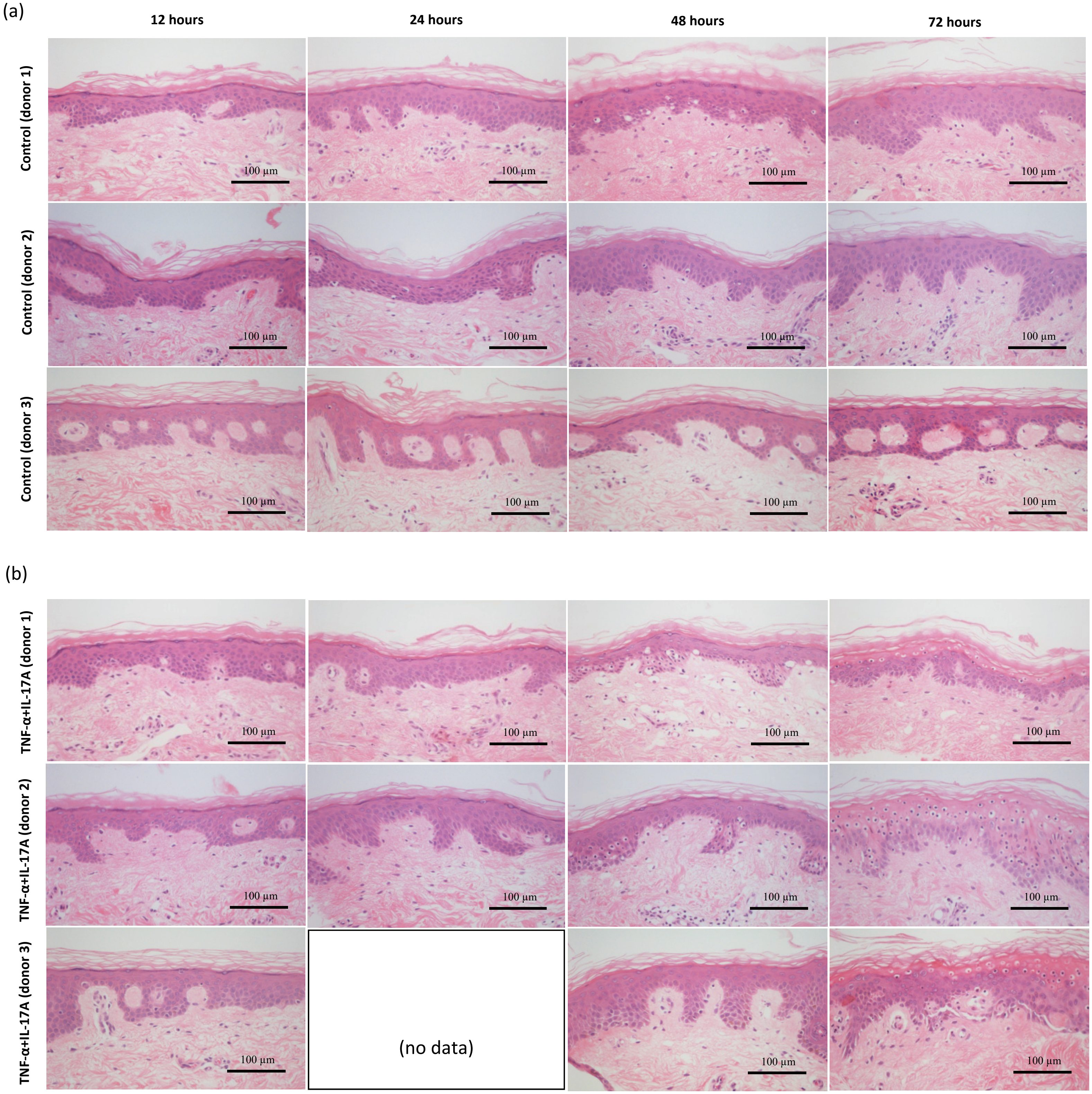

Supplemental Figure 1: Histology of normal human skin organ cultures. Unstimulated skin organ cultures (a), 10ng ml^{-1} TNF + 20 ng ml^{-1} IL-17A treated skin organ cultures (b). 12-72 hour time course, 3 healthy donors, Scale bar, 100 μm.
