# Supplementary material for: The small molecule rhodomyrtone suppresses TNF-α and IL-17A-induced keratinocyte inflammatory responses: A potential new therapeutic for psoriasis
Source: PLoS One. 2018 Oct 15;13(10):e0205340. doi: 10.1371/journal.pone.0205340 (PMC6188632; doi:10.1371/journal.pone.0205340)
Supplement: S2 Fig — Cultures of primary normal human keratinocytes (NHK) were treated with the indicated concentrations of rhodomyrtone or 5μM erlotinib for 24 hours and the level of cell death assessed using 4',6‐diamidino‐2‐phenylindole (DAPI) to identify the sub‐G1 cell cycle population (a and b) and annexin V‐FITC and propidium iodide (PI) staining to identify apoptotic and necrotic cells (c and d) using flow cytometry. Treatment with the EGFR inhibitor erlotinib, a positive control for keratinocyte apoptosis, increased the proportion of keratinocytes in the sub‐G1 (9.8%) as did treatment with 800 and 1,000 ng ml‐1 of rhodomyrtone (27.86% and 37.26%, respectively compared with 2.43% in control cultures); however, at concentrations of 600 ng/ml and below, rhodomyrtone did not exhibit toxicity (a and b). This result was corroborated using annexin V and PI staining which showed that below 600 ng/ml, rhodomyrtone did not induce keratinocyte apoptosis, however, cell death was evident at higher concentrations (c and d). Bar graphs, mean±SD of results from three independent experiments in triplicate. Statistical significance determined using one‐way ANOVA and Dunn’s multiple comparison test and denoted ** p<0.01, *** p<0.001, ****, p<0.0001. (PDF) [file pone.0205340.s002.pdf]

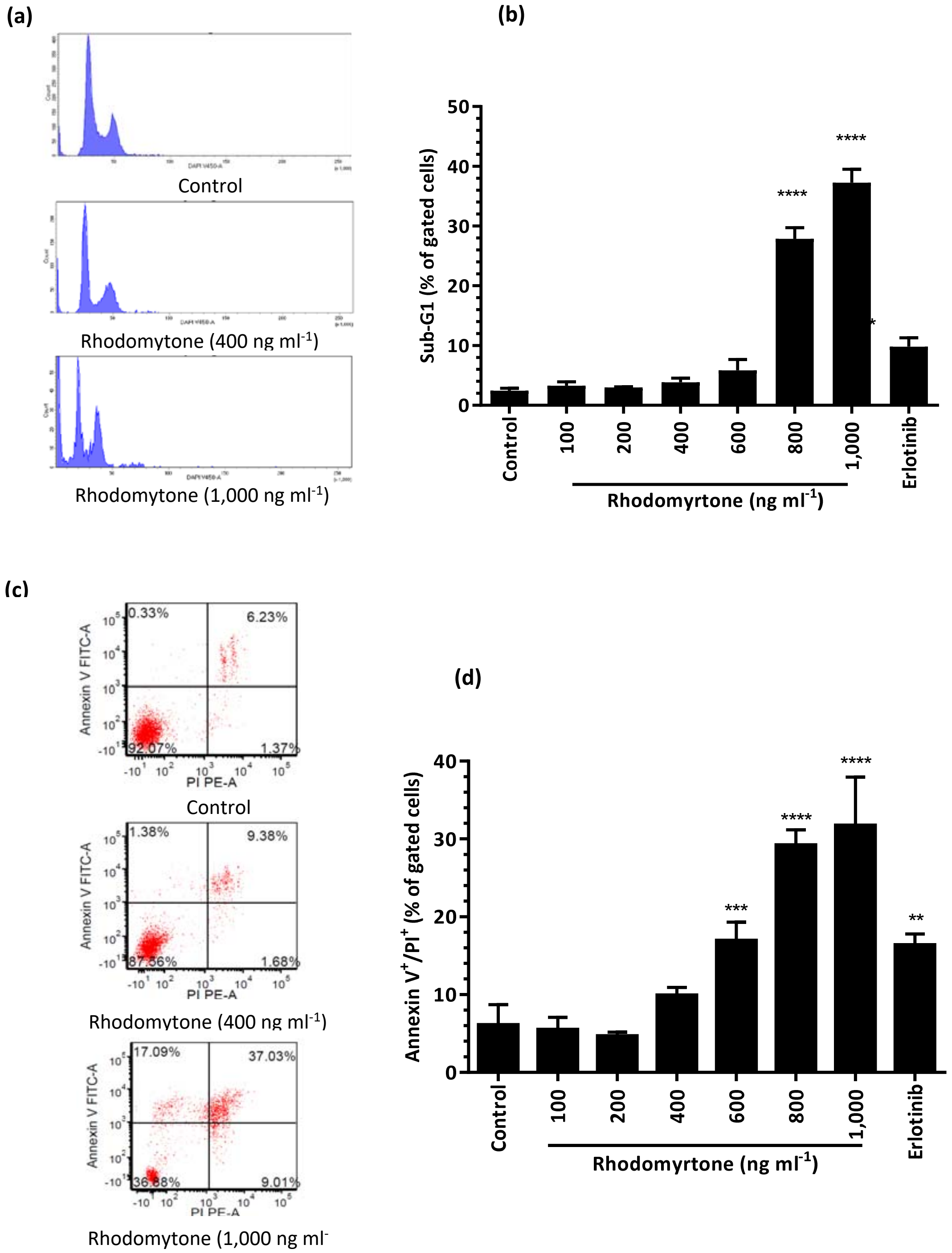

Supplemental Figure 2: Rhodomyrtone toxicity is limited to high doses. Cultures of primary normal human keratinocytes (NHK) were treated with the indicated concentrations of rhodomyrtone or 5µM erlotinib for 24 hours and the level of cell death assessed using 4',6-diamidino-2-phenylindole (DAPI) to identify the sub-G_1 cell cycle population (a and b) and annexin V-FITC and propidium iodide (PI) staining to identify apoptotic and necrotic cells (c and d) using flow cytometry. Treatment with the EGFR inhibitor erlotinib, a positive control for keratinocyte apoptosis [45], increased the proportion of keratinocytes in the sub-G_1 (9.8%) as did treatment with 800 and 1,000 ng ml^{-1} of rhodomyrtone (27.86% and 37.26%, respectively compared with 2.43% in control cultures); however, at concentrations of 600 ng ml^{-1} and below, rhodomyrtone did not exhibit toxicity (a and b). This result was corroborated using annexin V and PI staining which showed that below 600 ng ml^{-1}, rhodomyrtone did not induce keratinocyte apoptosis, however, cell death was evident at higher concentrations (c and d). Bar graphs, mean±SD of results from three independent experiments in triplicate. Statistical significance determined using one-way ANOVA and Dunn's multiple comparison test and denoted ** $p<0.01$, *** $p<0.001$, ****, $p<0.0001$.
