# Supplementary material for: The small molecule rhodomyrtone suppresses TNF-α and IL-17A-induced keratinocyte inflammatory responses: A potential new therapeutic for psoriasis
Source: PLoS One. 2018 Oct 15;13(10):e0205340. doi: 10.1371/journal.pone.0205340 (PMC6188632; doi:10.1371/journal.pone.0205340)
Supplement: S3 Fig — Cultures were pre-treated with 400ng ml-1 rhodomyrtone for 6 hours before stimulation with 10ng/ml TNF-α and 20ng/ml IL-17A for 72 hours. Immuno-histochemical staining for HBD-2, IL-36γ, and S100A7 revealed prominent induction of HBD-2 (a), IL-36G (b) and S100A7 (c) by TNF+IL17A treatment. Staining from 4 donors of 5 shown. Scale bar, 100 μm. (PDF) [file pone.0205340.s003.pdf]

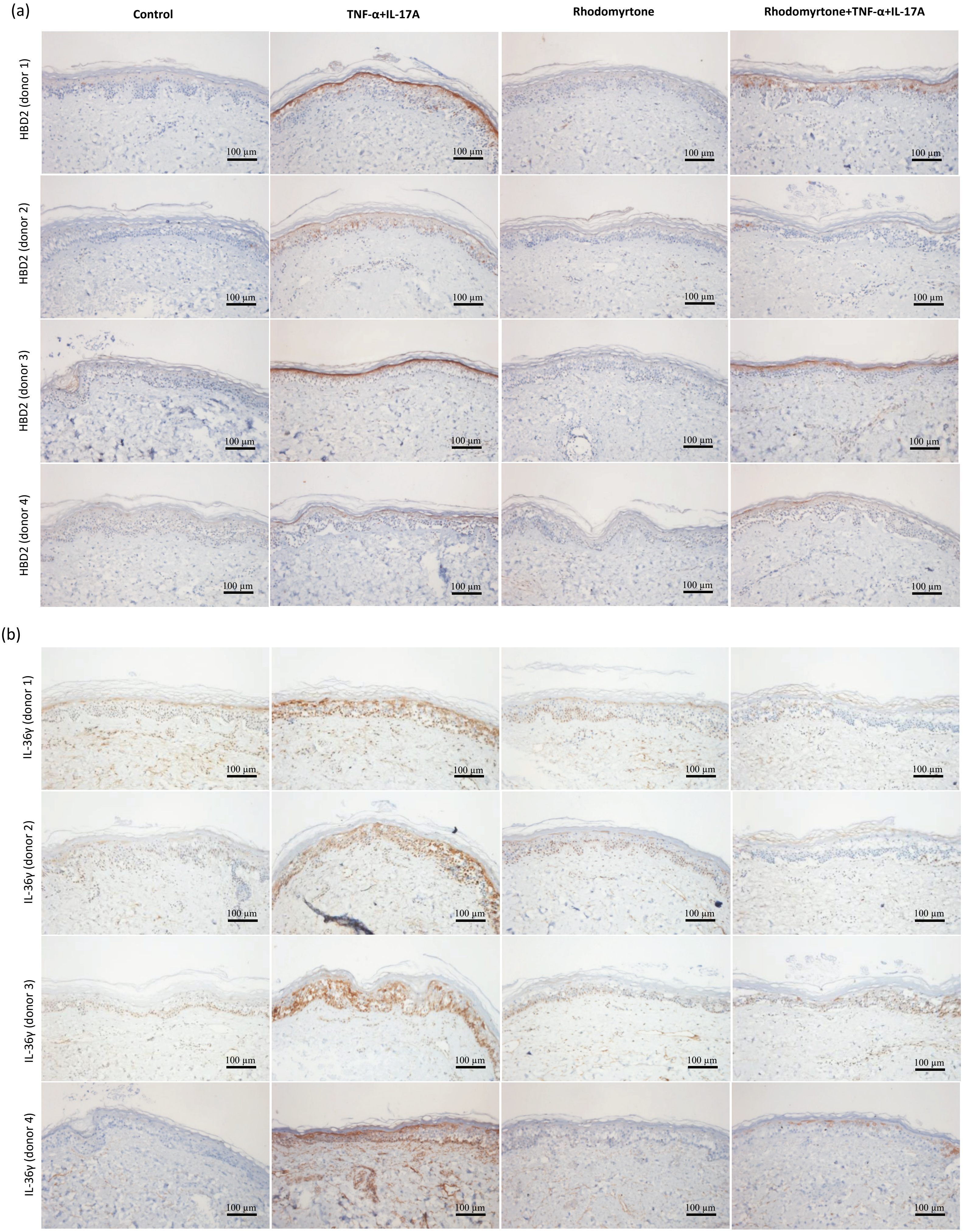
(a)
Control
TNF-α+IL-17A
Rhodomyrtone
Rhodomyrtone+TNF-α+IL-17A
HBD2 (donor 1)
HBD2 (donor 2)
HBD2 (donor 3)
HBD2 (donor 4)
100 µm
(b)
IL-36γ (donor 1)
IL-36γ (donor 2)
IL-36γ (donor 3)
IL-36γ (donor 4)

(c)

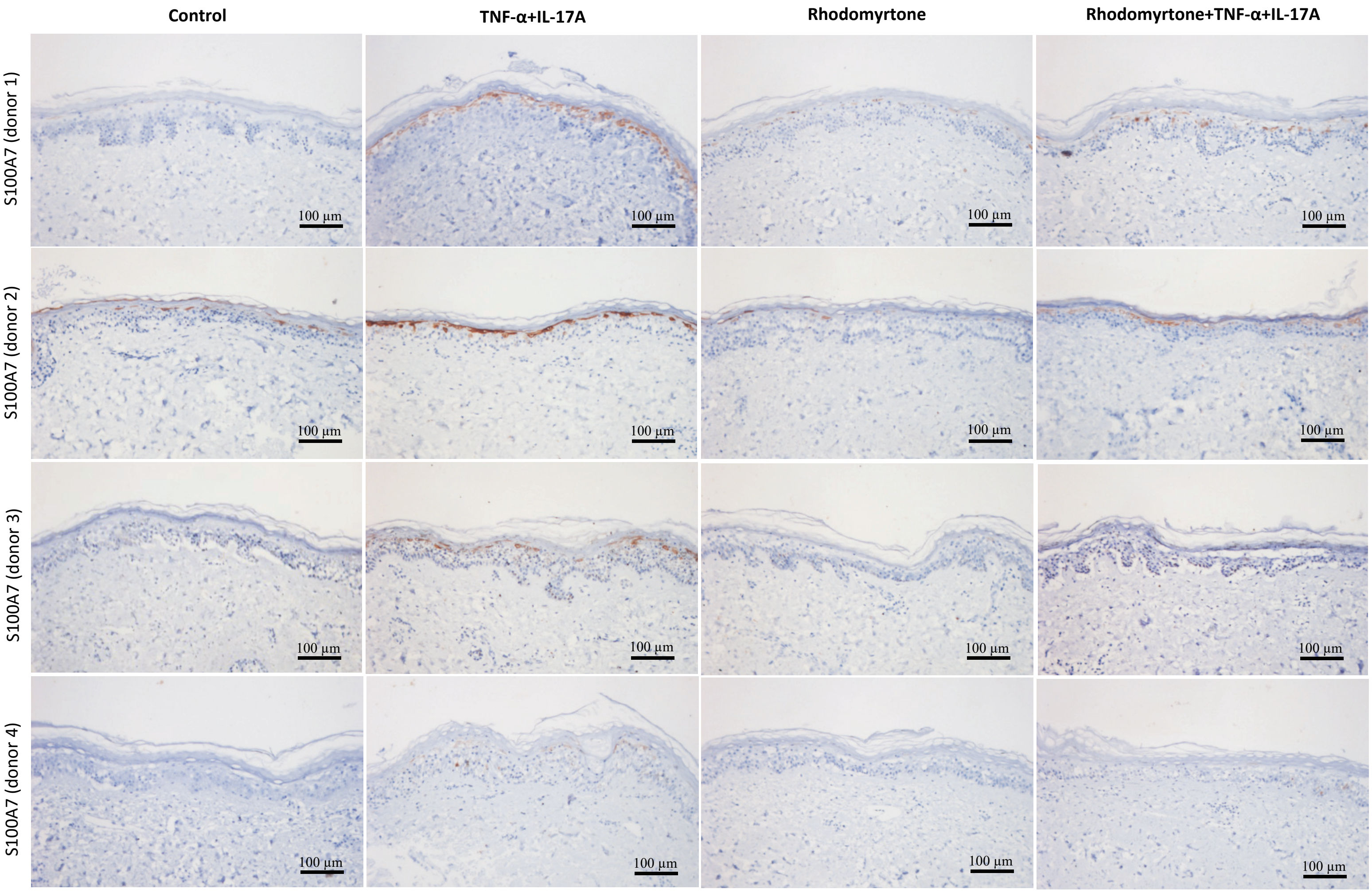

Supplemental Figure 3: Rhodomyrtone inhibits TNF-α+IL-17A-induced inflammatory responses in skin organ cultures. Cultures were pre-treated with 400ng ml^{-1} rhodomyrtone for 6 hours before stimulation with 10ng ml^{-1} TNF-α and 20ng ml^{-1} IL-17A for 72 hours. Immuno-histochemical staining for HBD-2, IL-36γ, and S100A7 revealed prominent induction of HBD-2 (a), IL-36G (b) and S100A7 (c) by TNF+IL17A treatment. Staining from 4 donors of 5 shown. Scale bar, 100 µm.
