# Supplementary material for: The small molecule rhodomyrtone suppresses TNF-α and IL-17A-induced keratinocyte inflammatory responses: A potential new therapeutic for psoriasis
Source: PLoS One. 2018 Oct 15;13(10):e0205340. doi: 10.1371/journal.pone.0205340 (PMC6188632; doi:10.1371/journal.pone.0205340)
Supplement: S4 Fig — Mice received a daily topical dose of 62.5 mg IMQ cream (5% on the shaved dorsal skin for 15 days. Six days after first sensitization, IMQ‐induced mice were treated with rhodomyrtone formulations (0.181 and 0.364 mg/cm), or vehicle group, or betamethasone (0.015 mg/cm) twice a day for 9 consecutive days. At 24 hours after the final administration, mice were sacrificed and samples were taken. (PDF) [file pone.0205340.s004.pdf]

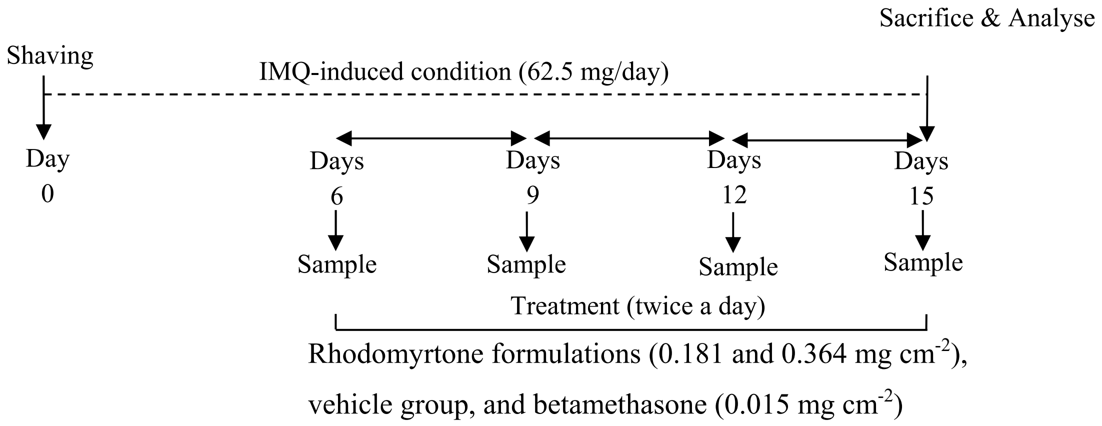

Supplemental Figure 4: Experimental procedure of IMQ-induced skin-inflammation in mice. Mice received a daily topical dose of 62.5 mg IMQ cream (5% on the shaved dorsal skin for 15 days. Six days after first sensitization, IMQ-induced mice were treated with rhodomyrtone formulations (0.181 and 0.364 mg cm^{-2}), or vehicle group, or betamethasone (0.015 mg cm^{-2}) twice a day for 9 consecutive days. At 24 hours after the final administration, mice were sacrificed and samples were taken.
