# Supplementary material for: The small molecule rhodomyrtone suppresses TNF-α and IL-17A-induced keratinocyte inflammatory responses: A potential new therapeutic for psoriasis
Source: PLoS One. 2018 Oct 15;13(10):e0205340. doi: 10.1371/journal.pone.0205340 (PMC6188632; doi:10.1371/journal.pone.0205340)
Supplement: S5 Fig — The control mice showed no changes in skin lesions (a). The mice received a daily topical dose of 62.5 mg of 5% IMQ cream demonstrate gross skin folds scaling, thickness, and erythema (b). Some decrease in scaling, thickness, and erythema were noticed in mice skin after treatment with base formulation (C). Rhodomyrtone formulation with 0.181 mg/cm (d), 0.364 mg/cm (e), and betamethasone cream with 0.015 mg/cm (f) demonstrated almost complete clearance of scaling, thickness, and erythema on the skin. (PDF) [file pone.0205340.s005.pdf]

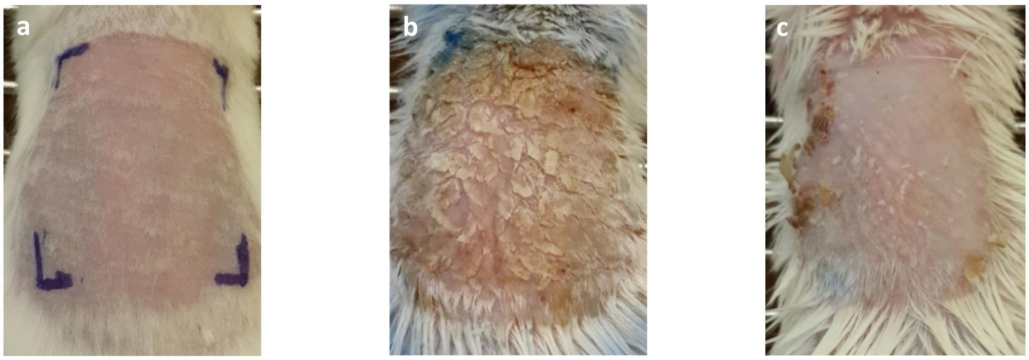

Control | IMQ | IMQ + base formulation

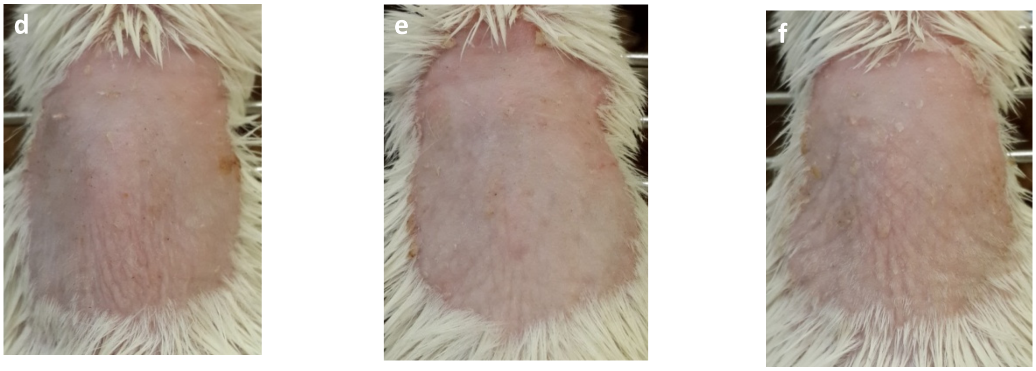

IMQ + rhodomyrtone formulation (0.181 mg cm^{-2}) | IMQ + rhodomyrtone formulation (0.364 mg cm^{-2}) | IMQ + betamethasone (0.015 mg cm^{-2})

Supplemental Figure 5: Rhodomyrtone attenuates imiquimod (IMQ)-induced skin inflammation in mice. The control mice showed no changes in skin lesions (a). The mice received a daily topical dose of 62.5 mg of 5% IMQ cream demonstrate gross skin folds scaling, thickness, and erythema (b). Some decrease in scaling, thickness, and erythema were noticed in mice skin after treatment with base formulation (C). Rhodomyrtone formulation with 0.181 mg cm^{-2} (d), 0.364 mg cm^{-2} (e), and betamethasone cream with 0.015 mg cm^{-2} (f) demonstrated almost complete clearance of scaling, thickness, and erythema on the skin.
