# Supplementary material for: The small molecule rhodomyrtone suppresses TNF-α and IL-17A-induced keratinocyte inflammatory responses: A potential new therapeutic for psoriasis
Source: PLoS One. 2018 Oct 15;13(10):e0205340. doi: 10.1371/journal.pone.0205340 (PMC6188632; doi:10.1371/journal.pone.0205340)
Supplement: S6 Fig — HaCaT keratinocytes were treated with 2μg ml-1 rhodomyrtone for 0, 12, 24, 48, and 72 hours then total protein prepared. The effects of rhodomyrtone on expression levels of apoptosis-associated proteins was assesses by western blot analyses using antibodies against Bcl-2, Bid, caspases-7, -8, and -9, and cleaved caspases-7, -8, and -9 (A). Bar graphs represent the ratio of each protein normalized to β-actin expression (B). Values shown are mean ± SD of results from three independent experiments in triplicate. (*p<0.05, **p<0.01, ****p<0.0001, as compared with control). (PDF) [file pone.0205340.s006.pdf]

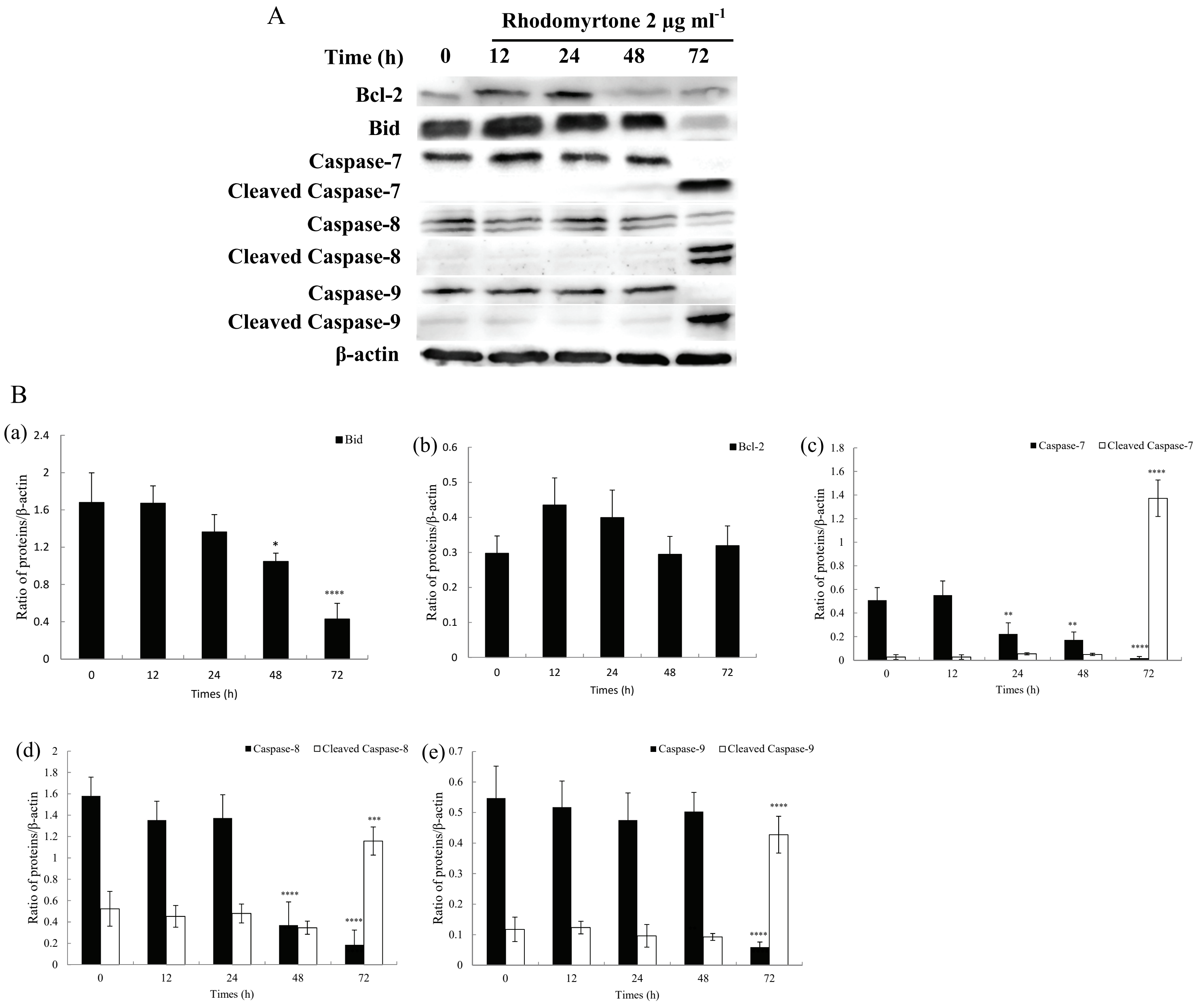

Supplemental Figure 6: At high concentrations, rhodomyrtone triggers activation of keratinocyte apoptosis. HaCaT keratinocytes were treated with 2μg ml^{-1} rhodomyrtone for 0, 12, 24, 48, and 72 hours then total protein prepared. The effects of rhodomyrtone on expression levels of apoptosis-associated proteins was assesses by western blot analyses using antibodies against Bcl-2, Bid, caspases-7, -8, and -9, and cleaved caspases-7, -8, and -9 (A). Bar graphs represent the ratio of each protein normalized to β-actin expression (B). Values shown are mean ± SD of results from three independent experiments in triplicate. ($^{*}p<0.05$, $^{**}p<0.01$, $^{****}p<0.0001$, as compared with control).
