# Supplementary material for: The small molecule rhodomyrtone suppresses TNF-α and IL-17A-induced keratinocyte inflammatory responses: A potential new therapeutic for psoriasis
Source: PLoS One. 2018 Oct 15;13(10):e0205340. doi: 10.1371/journal.pone.0205340 (PMC6188632; doi:10.1371/journal.pone.0205340)
Supplement: S1 Method — (PDF) [file pone.0205340.s008.pdf]

SUPPLEMENTAL METHODS

Apoptosis assay and cell-cycle analysis

Cells at a density of 4×10^4 cells $well^{-1}$ were seeded into 12-well plates and incubated at 37˚C for 24 hours. Rhodomyrtone (100-1,000 ng ml^{-1}) was added for 24 hours, after which the adherent cells were washed twice with PBS, trypsinized, and collected by centrifugation at 200x*g* for 4 minutes at 4˚C. Cells were fixed and permeabilized with 3 ml cold 70% ethanol at -20˚C overnight before centrifugation at 1500x*g* for 10 minutes. After washing, the cell pellets were incubated with 0.5 ml of PIPES buffer (10 mM Tris, 0.1 M NaCl, 2 mM $MgCl_2$, 0.1% Triton X-100, pH 6.8) and 1 µg ml^{-1} DAPI at RT for 10 minutes. Cell cycle and sub-G_1 distributions were analyzed by measuring DNA content using an LSR2 flow cytometer (Beckton-Dickinson). A second fraction of cells was used for the quantification of apoptosis and necrosis using FITC-annexin V and propidium iodide staining, followed by flow cytometric analysis.
